# Secondary Intracranial Hypertension During ATRA/ATO Therapy in High-Risk Acute Promyelocytic Leukemia: A Case Report and Practical Management Framework

**DOI:** 10.3390/curroncol33100611

**Published:** 2026-10-10

**Authors:** Luca Roeh, Nigel Schaeper, Sebastian Schröder, Jan Friedrich Scheitz, Dorothea Cornelia Voigt, Simon Schallenberg, Martin Janz, Philipp le Coutre, Sebastian Stintzing, Daniel Noerenberg

**Affiliations:** 1 Department of Hematology, Oncology and Tumor Immunology, Charité Campus Mitte, Charité–Universitätsmedizin Berlin, Corporate Member of Freie Universität Berlin and Humboldt-Universität zu Berlin, 10117 Berlin, Germany; 2 Department of Internal Medicine, Bundeswehr Hospital Berlin, 10115 Berlin, Germany; 3 Department of Neurology with Experimental Neurology and Center for Stroke Research Berlin, Charité–Universitätsmedizin Berlin, Corporate Member of Freie Universität Berlin and Humboldt-Universität zu Berlin, 10117 Berlin, Germany; 4 Institute of Neuroradiology, Charité–Universitätsmedizin Berlin, Corporate Member of Freie Universität Berlin and Humboldt-Universität zu Berlin, 10117 Berlin, Germany; 5 Institute of Pathology, Charité–Universitätsmedizin Berlin, Corporate Member of Freie Universität Berlin and Humboldt-Universität zu Berlin, 10117 Berlin, Germany; 6 Department of Hematology, Oncology and Tumor Immunology, Campus Virchow-Klinikum, Charité–Universitätsmedizin Berlin, Corporate Member of Freie Universität Berlin and Humboldt-Universität zu Berlin, 13353 Berlin, Germany; 7 Research Group Biology of Malignant Lymphomas, Max Delbrück Center for Molecular Medicine in the Helmholtz Association, 13125 Berlin, Germany

**Keywords:** acute promyelocytic leukemia, all-trans retinoic acid, arsenic trioxide, secondary intracranial hypertension, pseudotumor cerebri, papilledema, acetazolamide, consolidation therapy

## Abstract

All-trans retinoic acid (ATRA) combined with arsenic trioxide (ATO) is the standard of care for acute promyelocytic leukemia (APL), including high-risk disease. Secondary intracranial hypertension (SIH), historically termed pseudotumor cerebri (PTC), is a recognized but uncommon and potentially therapy-limiting complication of retinoid exposure, reported predominantly in pediatric and young-adult patients and most frequently during induction. We report a 31-year-old man with high-risk microgranular APL in whom SIH was diagnosed during the first ATRA/ATO consolidation cycle. The clinical course included progressive headache, ocular pain, pulsatile tinnitus, focusing difficulties, visual deterioration, and diplopia. Ophthalmological assessment demonstrated bilateral papilledema with marked peripapillary retinal nerve fiber layer thickening despite preserved central visual acuity. Magnetic resonance imaging showed bilateral optic nerve sheath distension, prominent optic nerve heads, mild pituitary flattening, and bilateral transverse sinus stenosis. Lumbar puncture demonstrated an opening pressure of 32 cm H_2_O with otherwise unremarkable cerebrospinal fluid; removal of approximately 30 mL resulted in immediate symptomatic improvement. Management consisted of temporary ATRA interruption, therapeutic lumbar punctures, acetazolamide, and individualized ATRA rechallenge and dose titration. Repeated attempts at dose escalation reproduced symptoms, and ATRA was ultimately maintained at a maximally tolerated dose of 30 mg/m^2^/day while full-dose ATO was continued. Molecular remission was achieved after the first consolidation cycle and remains sustained at 18-month follow-up. This case highlights the importance of recognizing ATRA-associated SIH throughout the treatment course and illustrates a pragmatic strategy for preserving ATRA exposure when standard-dose treatment is limited by neurological toxicity. However, the oncological equivalence of reduced-dose ATRA cannot be inferred from a single case.

## Simple Summary

Acute promyelocytic leukemia is an aggressive blood cancer that can now be cured in most patients with a combination of a vitamin-A-derived drug and arsenic trioxide. We describe a young adult who developed a rare side effect in which the pressure of the fluid surrounding the brain rises, causing headache, ringing in the ears, and visual disturbances. Although one major clinical trial reported a single event of increased intracranial pressure, detailed information on its diagnosis and management was not provided. By briefly pausing the vitamin-A-derived drug, draining small amounts of spinal fluid, adding a medication that reduces the production of brain fluid, and carefully reducing the drug dose, we controlled the symptoms while continuing curative treatment, and the patient remains cancer-free. When new neurological symptoms appear during therapy, physicians should consider this treatable side effect after ruling out other serious causes such as infection of the central nervous system, bleeding, blood clots in the brain’s veins, and a return or progression of the leukemia.

## 1. Introduction

All-trans retinoic acid (ATRA) and arsenic trioxide (ATO) have transformed acute promyelocytic leukemia (APL) from a highly fatal subtype of acute myeloid leukemia into one of the most curable hematological malignancies. ATRA induces differentiation of leukemic promyelocytes by overcoming transcriptional repression mediated by *PML::RARA,* whereas ATO promotes degradation of the *PML::RARA* oncoprotein and apoptosis of APL cells. Combination strategies incorporating ATRA and ATO have consequently become central to modern APL treatment [1,2,3,4]. More recently, the randomized phase III APOLLO trial provided evidence supporting an ATRA/ATO-based strategy incorporating limited anthracycline exposure in newly diagnosed high-risk APL [1].

Despite its therapeutic importance, ATRA is associated with characteristic toxicities, including differentiation syndrome, hepatotoxicity, hyperlipidemia, and neurological adverse effects [3]. SIH represents an uncommon but clinically important retinoid-associated toxicity. The syndrome is characterized by increased intracranial pressure in the absence of an intracranial mass lesion, hydrocephalus, or another structural explanation and commonly presents with headache, pulsatile tinnitus, transient visual disturbances, diplopia, and papilledema [5,6,7]. Vitamin A derivatives are among the drugs most strongly associated with drug-induced intracranial hypertension [7].

ATRA-associated SIH has historically been described particularly in children, adolescents, and young adults and frequently develops during the first weeks of induction therapy [8,9,10,11,12,13]. In the pediatric GIMEMA-AIEOPAIDA study, pseudotumor cerebri occurred in 10 of 107 evaluable children [8]. In an analysis of Intergroup Protocol 0129, probable PTC occurred in 4 of 240 ATRA-exposed patients (1.7%), with a higher incidence in the pediatric population [10]. Adult cases nevertheless occur, and published reports demonstrate that intracranial hypertension may occur outside the initial induction period [11,12,13,14,15,16,17]. Thus, this complication should neither be regarded as exclusively pediatric nor restricted to early ATRA exposure.

Management poses a particular challenge because neurological toxicity must be controlled promptly to protect visual function while ATRA remains an important component of curative-intent therapy. Published approaches include temporary ATRA interruption or dose reduction, therapeutic cerebrospinal fluid (CSF) drainage, acetazolamide, topiramate, corticosteroids, and, in refractory cases, CSF shunting [9,11,12,13,14,15,17]. Smith et al., for example, described ATRA interruption or modification together with acetazolamide or topiramate in patients receiving ATRA/ATO [11]. More recently, CSF shunting has been used in a refractory case to facilitate continued ATRA administration [17]. However, no standardized management exists, and evidence regarding ATRA rechallenge and the oncological consequences of reduced cumulative exposure remains limited.

Here, we report a 31-year-old man with high-risk microgranular APL in whom clinically significant SIH was diagnosed during the first ATRA/ATO consolidation cycle. The case is characterized by objectively documented papilledema, radiological features of increased intracranial pressure, elevated CSF opening pressure, reproducible symptom recurrence during ATRA dose escalation, and successful completion of ATRA/ATO consolidation using prolonged intracranial-pressure-lowering therapy and individualized ATRA dose titration. We additionally provide a focused review of the available literature and propose a practical management framework while emphasizing the limitations of the current evidence.

## 2. Detailed Case Description

**Figure 1:**
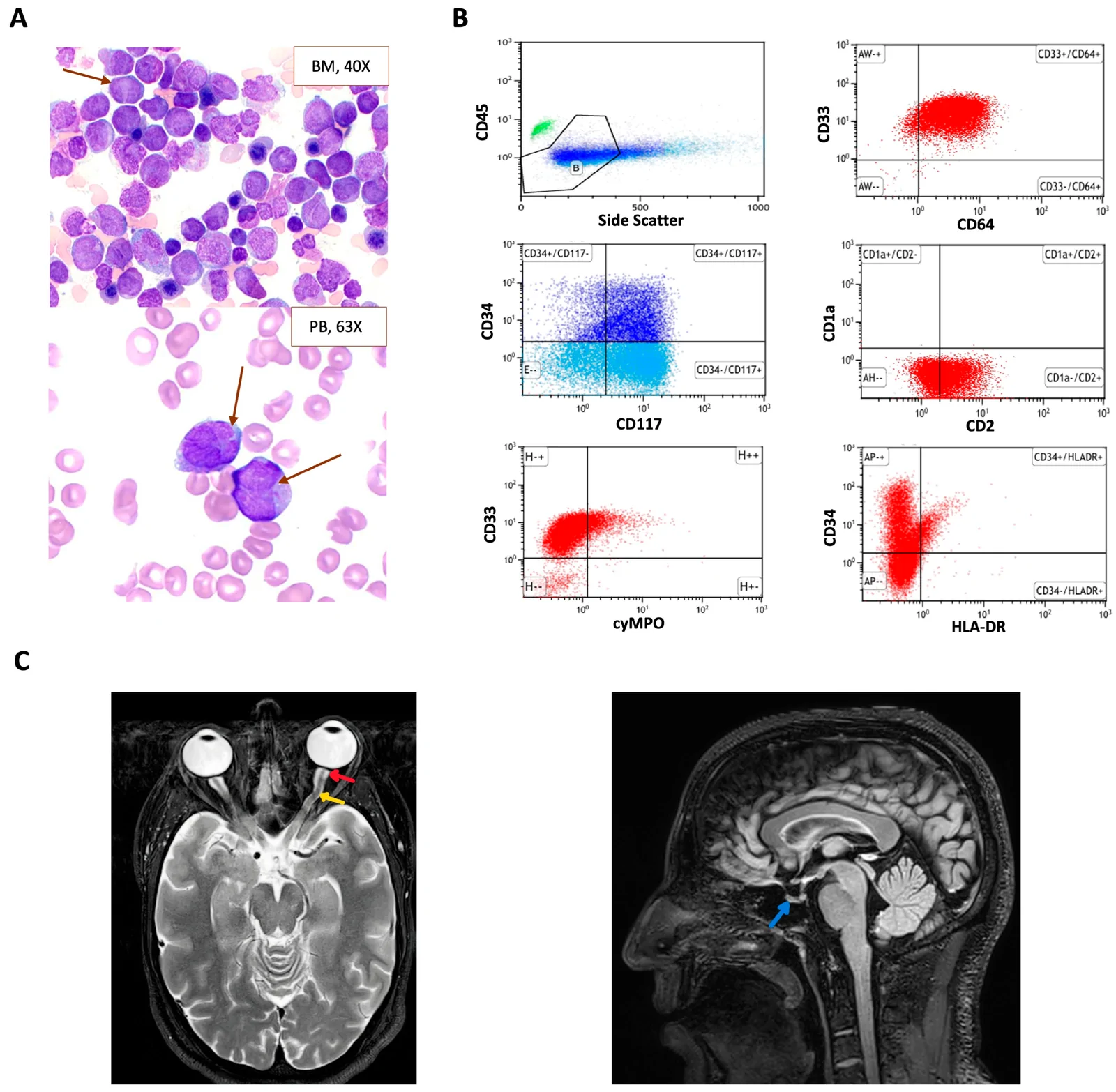
Diagnostic work-up of a 31-year-old patient with high-risk APL at diagnosis and during neurotoxicity under therapy with ATRA/ATO. (**A**) Bone marrow aspirate (upper, panoptic stain, 40×) and peripheral blood smear (lower, panoptic stain, 63×) showing abnormal promyelocytes, monocytic blasts with bilobed nuclei (red arrow). No Auer rods were seen. (**B**) Multiparameter flow cytometry of the dominant leukemic population with the phenotype CD34^+^CD117^+^CD13^+^CD33^+^CD64^+^cyMPO^dim^CD2^+^HLA-DR^−^. (**C**) Brain magnetic resonance imaging (MRI) at consolidation cycle I with ATRA/ATO showing signs of secondary intracranial hypertension (SIH). Arrows indicate accentuated optic nerve sheath (yellow arrow) and prominent optic nerve heads (red arrow), as well as a flattened pituitary gland (blue arrow).

A 31-year-old man presented to our emergency department in March 2025 with a two-week history of recurrent epistaxis and petechiae of the oral mucosa. Laboratory evaluation revealed a white blood cell count of 28 × 10^9^/L with 0.4 × 10^9^/L neutrophils, a platelet count of 10 × 10^9^/L, hemoglobin of 10.7 g/dL, prolonged coagulation parameters, decreased fibrinogen, markedly elevated D-dimers, and lactate dehydrogenase of 580 U/L. Peripheral blood smear demonstrated normochromic, normocytic anemia, severe thrombocytopenia, and more than 90% circulating monocytoid blast-like cells. Bone marrow aspiration showed approximately 70% infiltration by blastoid cells without the classical appearance of hypergranular promyelocytes or Auer rods, raising suspicion for the microgranular variant of APL (M3v) (Figure 1A). Flow cytometry demonstrated a dominant leukemic population expressing CD34, CD117, CD13, CD33, CD64, and CD2 in the absence of HLA-DR and lymphoid markers (Figure 1B).

Given the clinical suspicion of APL and associated risk of fatal hemorrhage, ATRA and hydroxyurea were initiated within 24 h of hospital admission while confirmatory diagnostics were expedited. Prompt molecular testing demonstrated a *PML::RARA* fusion transcript, bcr-3 isoform, with additional *FLT3-*ITD and *FLT3*-TKD mutations. Cytogenetic analysis confirmed t(15;17). With a presenting leukocyte count exceeding 10 × 10^9^/L, the patient was classified as having high-risk APL.

At initial APL diagnosis, the patient was 175 cm tall and weighed 93 kg, corresponding to a BMI of 30.4 kg/m^2^. The patient had a muscular body habitus and no relevant past medical history. Body weight remained essentially stable throughout treatment without substantial weight gain. Concomitant medication at the time of SIH consisted of amlodipine 10 mg once daily, pantoprazole 40 mg once daily, and acyclovir 400 mg twice daily. No concomitant tetracycline, azole antifungal, or additional vitamin A/retinoid-containing medication or supplement was documented.

Induction therapy consisted of ATRA 45 mg/m^2^/day in two divided doses combined with idarubicin 12 mg/m^2^ on days 1, 3, 5, and 7. Prednisolone at 1 mg/kg body weight was administered for differentiation-syndrome prophylaxis. Intensive supportive care included fresh frozen plasma, fibrinogen, and platelet transfusions to maintain hemostatic targets. ATO was subsequently introduced at 0.15 mg/kg/day after initial cytoreduction. The patient did not develop differentiation syndrome or major hemorrhage and achieved morphological complete remission by day 28. Residual disease remained detectable by multiparameter flow cytometry and quantitative polymerase chain reaction (qPCR), and ATRA/ATO consolidation was initiated as planned.

Approximately 3–4 weeks before neurological admission in May 2025, the patient had experienced severe predominantly nocturnal headache accompanied by ocular pain, pulsatile tinnitus, and photophobia. Although these symptoms initially improved, difficulty focusing persisted and was followed by gradually progressive subjective visual deterioration. Approximately 3–4 days before neurological presentation, the patient additionally developed diplopia at near fixation and a subjective sensation of strabismus. Thus, the first neurological symptoms preceded definitive diagnosis, whereas SIH was diagnosed during the first ATRA/ATO consolidation cycle.

Neurological examination demonstrated subjective diplopia on lateral gaze, more pronounced to the right, and bilateral retrobulbar headache without other focal neurological deficits.

Ophthalmological assessment on 13 May 2025, demonstrated bilateral papilledema. Despite marked structural optic disc swelling, uncorrected visual acuity remained 1.0 in both eyes. Gross confrontation visual-field testing was unremarkable. Pupils were equal and reactive, and no relative afferent pupillary defect was present. Fundus examination and photography documented optic disc swelling. Optical coherence tomography (OCT) demonstrated pronounced bilateral peripapillary retinal nerve fiber layer (RNFL) thickening, with an average RNFL thickness of 336 µm in the right eye and 230 µm in the left eye. A formal Frisén grade was not documented at presentation and was therefore not assigned retrospectively.

An earlier non-contrast brain MRI had not identified a relevant structural abnormality. Following recognition of papilledema, native cranial computed tomography was likewise non-contributory. Subsequent MRI demonstrated bilateral distension of the optic nerve sheaths, prominent optic nerve heads, mild flattening of the pituitary gland, and bilateral transverse sinus stenosis (Figure 1C). There was no intracranial hemorrhage, mass lesion, obstructive hydrocephalus, or cerebral venous sinus thrombosis.

The initial diagnostic lumbar puncture was performed in the lateral decubitus position without sedation and revealed an elevated opening pressure of 32 cm H_2_O. Approximately 30 mL of clear CSF were removed, resulting in immediate improvement of pulsatile tinnitus and marked improvement of the pressure-associated symptoms. A closing pressure was not documented. CSF composition was unremarkable, and flow-cytometric analysis demonstrated no malignant cells. Subsequent contrast-enhanced MRI on 23 May 2025 showed no evidence of meningeal leukemic involvement.

Taken together, bilateral papilledema, MRI features associated with raised intracranial pressure, elevated CSF opening pressure, normal CSF composition, exclusion of relevant structural, vascular, infectious, and leukemic CNS pathology, and prompt improvement after CSF drainage supported the diagnosis of SIH. ATRA was considered the principal treatment-related contributor and was temporarily discontinued.

Acetazolamide was initiated on 14 May 2025, at 750 mg twice daily (1500 mg/day) to further reduce intracranial pressure, necessitating close monitoring and adjustment due to transient renal impairment and marked hypokalemia and hypomagnesemia, requiring substantial supplementation in the context of ATO exposure to mitigate the risk of cardiac adverse events. Following symptomatic improvement, ATRA was cautiously reintroduced at 50% of the initial dose while ATO was continued at full dose. Attempts to increase ATRA toward 30 mg twice daily (60 mg/day) repeatedly resulted within days in recurrent headache, pulsatile tinnitus, and ocular discomfort, despite escalation of acetazolamide to 750 mg three times daily (2250 mg/day).

Following another symptomatic recurrence, a second therapeutic lumbar puncture was performed on 3 July 2025, without sedation. In contrast to the initial diagnostic procedure, this lumbar puncture was performed with the patient in the sitting position. CSF flow appeared brisk, but opening pressure was not formally measured. Therapeutic drainage of 20 mL of CSF again resulted in rapid symptomatic improvement. Consequently, acetazolamide dose was further increased to 1000 mg three times per day (3000 mg/day).

To avoid repeated lumbar punctures while maintaining ATRA exposure, ATRA was restarted at 20 mg in the morning and 30 mg in the evening (50 mg/day) after the second lumbar puncture on 3 July 2025, and increased after one week to 30 mg twice daily (60 mg/day, approximately 30 mg/m^2^/day, about 67% of the standard adult dose), a level that had previously triggered symptom recurrence but was now tolerated. This dose was maintained for the remainder of consolidation. In agreement with the patient, no further escalation was attempted as low-grade headache and intermittent tinnitus persisted. Visual symptoms did not recur. Symptom burden fluctuated with treatment cycles, improving during treatment-free intervals and increasing during periods of combined ATRA/ATO exposure.

Despite these complications, full-dose ATO and dose-adjusted ATRA were continued through consolidation. qPCR demonstrated molecular remission after the first consolidation cycle, with undetectable *PML::RARA* transcripts in both bone marrow and peripheral blood.

ATRA/ATO treatment was completed in October 2025. During the same month, acetazolamide was reduced to 500 mg twice daily (1000 mg/day) and was discontinued completely on 18 November 2025. Neurological symptoms resolved almost completely within approximately four weeks after completion of ATRA/ATO treatment. At 18-month follow-up, the patient remains in molecular remission.

## 3. Discussion

This case describes clinically significant SIH associated with ATRA exposure in a young adult receiving ATRA/ATO-based treatment for high-risk APL. Several aspects are particularly informative: SIH was diagnosed outside the typical pediatric early-induction setting; substantial structural optic disc abnormalities were present despite preserved central visual acuity; symptom recurrence was reproducibly associated with attempts at ATRA dose escalation; and a combination of therapeutic CSF drainage, prolonged acetazolamide treatment, and individualized ATRA dose titration permitted completion of ATRA/ATO consolidation without permanent discontinuation of ATRA.

The clinical presentation evolved over several weeks. Headache, ocular pain, pulsatile tinnitus, photophobia, focusing difficulties, gradual subjective visual deterioration, and ultimately diplopia preceded recognition of bilateral papilledema. This spectrum is characteristic of intracranial hypertension and emphasizes that preserved central visual acuity does not exclude clinically important optic nerve involvement [5,6]. In our patient, visual acuity remained 1.0 bilaterally and no relative afferent pupillary defect was detected despite marked bilateral RNFL thickening on OCT.

The differential diagnosis in a patient undergoing treatment for high-risk APL is broad and potentially life-threatening. Intracranial hemorrhage, cerebral venous sinus thrombosis, CNS infection, hydrocephalus, intracranial mass lesions, leukemic CNS involvement, and other treatment-related neurotoxicities must be considered. The combination of papilledema, characteristic MRI findings, an opening pressure of 32 cm H_2_O, and symptomatic improvement after CSF drainage therefore strongly supported SIH.

Brain MRI may provide additional supportive evidence. Previously reported imaging findings include an empty or partially empty sella, posterior globe flattening, distension of the perioptic subarachnoid space, and transverse sinus stenosis [5,6,15,18]. Similar indirect signs were present in our patient, including bilateral optic nerve sheath distension, prominent optic nerve heads, mild pituitary flattening, and bilateral transverse sinus stenosis.

Lumbar puncture in a patient with suspected intracranial hypertension requires appropriate safety assessment. Neuroimaging should precede the procedure when a secondary cause or clinically relevant intracranial pressure gradient is suspected. In APL, thrombocytopenia, coagulopathy and local infection are additional procedural considerations. In our patient, relevant structural and vascular causes were excluded before CSF drainage.

For diagnostic assessment, opening pressure should ideally be obtained under standardized conditions. In the present case, the first quantitative measurement was obtained in the lateral decubitus position without sedation, whereas the second lumbar puncture was performed in the sitting position for therapeutic purposes and did not include formal pressure measurement. We therefore use only the first value of 32 cm H_2_O as quantitative evidence of raised intracranial pressure.

ATRA-associated SIH has been recognized since the early use of retinoids in APL, and selected reports are summarized in Table 1. Pediatric susceptibility was apparent in early reports and was subsequently confirmed in larger cohorts [8,10]. In the GIMEMA-AIEOPAIDA pediatric study, 10 of 107 evaluable children developed PTC [8]. Coombs et al. subsequently classified four of 240 ATRA-exposed patients in Intergroup Protocol 0129 as probable PTC, corresponding to 1.7%, and again observed greater susceptibility in pediatric patients [10].

Adult cases are nevertheless well documented. Sano et al. reported nausea, headache, diplopia, and bilateral papilledema after 23 days of ATRA treatment, with improvement following ATRA discontinuation and glycerol [9]. Anoop et al. subsequently reported ATRA-associated PTC during induction in an adult patient [13]. These observations support continued clinical vigilance beyond the classic pediatric phenotype.

Experience with combined ATRA/ATO is particularly relevant to contemporary practice. Smith et al. reported PTC in five of ten patients treated with ATRA/ATO at a single institution. The small sample size precludes extrapolation of this unusually high incidence, but the series is informative regarding management: temporary ATRA interruption or dose modification, acetazolamide, topiramate, and subsequent ATRA re-escalation were used [11]. The APOLLO trial reported a single serious adverse event coded as “intracranial pressure increased” in the ATRA/ATO arm (1/68 patients; 1.5%), suspected to be medication-related, but provided no detailed information on its diagnosis or management [1].

More recent reports confirm that the toxicity remains relevant in the ATRA/ATO era. Ramburuth and Khalil described ATRA-induced PTC in 2023, emphasizing early recognition and management to prevent visual loss [15]. Most recently, Creemers et al. described a 19-year-old woman with high-risk APL and recurrent SIH during induction and consolidation. Acetazolamide and reduced-dose ATRA reintroduction proved insufficient, and a lumboperitoneal shunt ultimately enabled continued ATRA administration and completion of consolidation [17].

Although ATRA-associated intracranial hypertension during consolidation has therefore been reported previously, including a recent abstract describing a similar dose-reduction strategy in low-/intermediate-risk APL [14], detailed evidence regarding management of recurrent symptoms during ATRA rechallenge in high-risk disease remains scarce. The particular contribution of the present case lies in the longitudinal documentation of an adult patient with high-risk APL, including quantitative neuro-ophthalmological findings, objectively elevated CSF opening pressure, immediate symptomatic responses to therapeutic CSF drainage, reproducible symptom recurrence during ATRA dose escalation, and subsequent symptom-guided ATRA titration to an individually tolerated dose while full-dose ATO was maintained.

### 3.1. Predisposing Factors and Potential Mechanisms

The pathophysiology of ATRA-associated SIH remains incompletely understood and is probably multifactorial. The association between retinoids and intracranial hypertension is biologically plausible given the established relationship between vitamin A derivatives and drug-induced intracranial hypertension [7]. Proposed mechanisms include altered CSF production or impaired CSF absorption, although the precise molecular pathways responsible for ATRA-associated disease remain uncertain.

Pharmacokinetic interactions may additionally modify individual susceptibility. Concomitant drugs capable of altering ATRA metabolism have been implicated in previous reports. This makes careful medication review particularly important. In the present patient, concomitant medication at the time of SIH only consisted of amlodipine, pantoprazole, and acyclovir.

Patient-related susceptibility should also be considered. Obesity is a recognized risk factor for idiopathic intracranial hypertension, although drug-induced intracranial hypertension can occur outside the classical demographic phenotype [5,6]. Our patient had a BMI of 30.4 kg/m^2^ at APL diagnosis but a muscular body habitus, and his weight remained essentially stable during treatment. BMI alone does not establish excess adiposity in this patient. Whether body composition contributed to SIH therefore remains uncertain.

The relationship between ATRA and SIH in the present patient should consequently be regarded as strongly suggestive rather than definitively causal. The temporal relationship with ATRA exposure, improvement following treatment interruption and CSF drainage, reproducible recurrence of headache, pulsatile tinnitus, and ocular discomfort during attempts at ATRA dose escalation, and improvement during treatment-free intervals support an ATRA-related contribution. Nevertheless, unmeasured individual susceptibility factors may also have contributed.

At present, no validated biomarker reliably predicts ATRA-associated SIH, and evidence is insufficient to support routine pharmacological prophylaxis. A practical preventive strategy therefore consists primarily of recognizing susceptibility factors, reviewing concomitant medication, and maintaining a low threshold for neurological and ophthalmological assessment when new headache, pulsatile tinnitus, diplopia, focusing difficulties, or visual symptoms occur during ATRA exposure.

### 3.2. Management and Preservation of Antileukemic Therapy

There is no standardized treatment strategy for ATRA-associated SIH. Immediate priorities are protection of visual function, reduction in intracranial pressure when indicated, and exclusion of secondary causes requiring specific treatment. Published approaches include temporary ATRA interruption, therapeutic lumbar puncture, acetazolamide, topiramate, corticosteroids, and individualized ATRA dose reduction or rechallenge [9,11,12,13,14,15,17]. In severe refractory disease with threatened visual function, CSF shunting may be considered.

Our case illustrates a stepwise individualized strategy. ATRA was initially interrupted, therapeutic CSF drainage produced rapid symptomatic improvement, and acetazolamide was initiated at 1500 mg/day. Once symptoms improved, ATRA was restarted at 50% of the initial dose. Repeated attempts at escalation toward standard-dose ATRA reproduced headache, pulsatile tinnitus, and ocular discomfort. Following a second therapeutic lumbar puncture, ATRA was gradually titrated to a maximally tolerated dose of 30 mg/m^2^/day while full-dose ATO was maintained.

Acetazolamide itself introduced clinically relevant management challenges. During treatment, transient renal impairment, hypokalemia, and hypomagnesemia required close laboratory surveillance and substantial supplementation. This deserves particular attention during concomitant ATO exposure because electrolyte abnormalities may increase susceptibility to QT prolongation and cardiac toxicity.

The optimal ATRA dose following development of SIH remains unknown. Children appear particularly susceptible to ATRA-associated intracranial hypertension, as noted above [8]. Lower ATRA doses have consequently been incorporated into pediatric treatment strategies, including ATRA 25 mg/m^2^/day in contemporary pediatric APL protocols [19]. These pediatric data cannot be directly extrapolated to adults and do not establish oncological equivalence between an individualized reduced dose and standard adult ATRA exposure.

Accordingly, the dose of 30 mg/m^2^/day in our patient should be understood exclusively as his individual maximally tolerated dose rather than a generally applicable treatment target. The therapeutic importance of maintaining effective differentiation therapy has become increasingly relevant as ATRA/ATO-based strategies have demonstrated excellent disease control across APL risk groups [1,2]. Nevertheless, preservation of antileukemic treatment intensity cannot take precedence over threatened visual function. Decisions regarding interruption, rechallenge, and dose escalation therefore require individualized assessment of neurological and ophthalmological severity, treatment phase, response to ICP-lowering measures, concomitant medications, and molecular disease response.

### 3.3. Limitations

Several limitations should be acknowledged. First, this is a single-patient observation and therefore cannot establish the safety, efficacy, or optimal dosing strategy of reduced-dose ATRA following SIH. Although molecular remission was achieved early and remains sustained at 18-month follow-up, this follow-up remains insufficient to exclude an increased long-term relapse risk resulting from reduced cumulative ATRA exposure. Accordingly, the observed response should not be interpreted as evidence that 30 mg/m^2^/day or any other reduced ATRA dose is oncologically equivalent to standard-dose therapy.

Second, although the temporal relationship with ATRA exposure, improvement following treatment interruption and CSF drainage, reproducible symptom recurrence during ATRA dose escalation, and fluctuation with treatment cycles support a drug-related association, causality cannot be established. Although the patient’s BMI was 30.4 kg/m^2^, his muscular body habitus and the absence of body-composition measurements limit its interpretation as evidence of excess adiposity or an additional susceptibility factor.

Third, although the initial diagnostic opening pressure was quantitatively documented at 32 cm H_2_O in the lateral decubitus position, opening pressure was not formally quantified at the second, therapeutic lumbar puncture. In addition, not all neuro-ophthalmological parameters were prospectively obtained according to a standardized SIH protocol. Formal Frisén grading was not documented and was therefore not assigned retrospectively.

**Figure 2:**
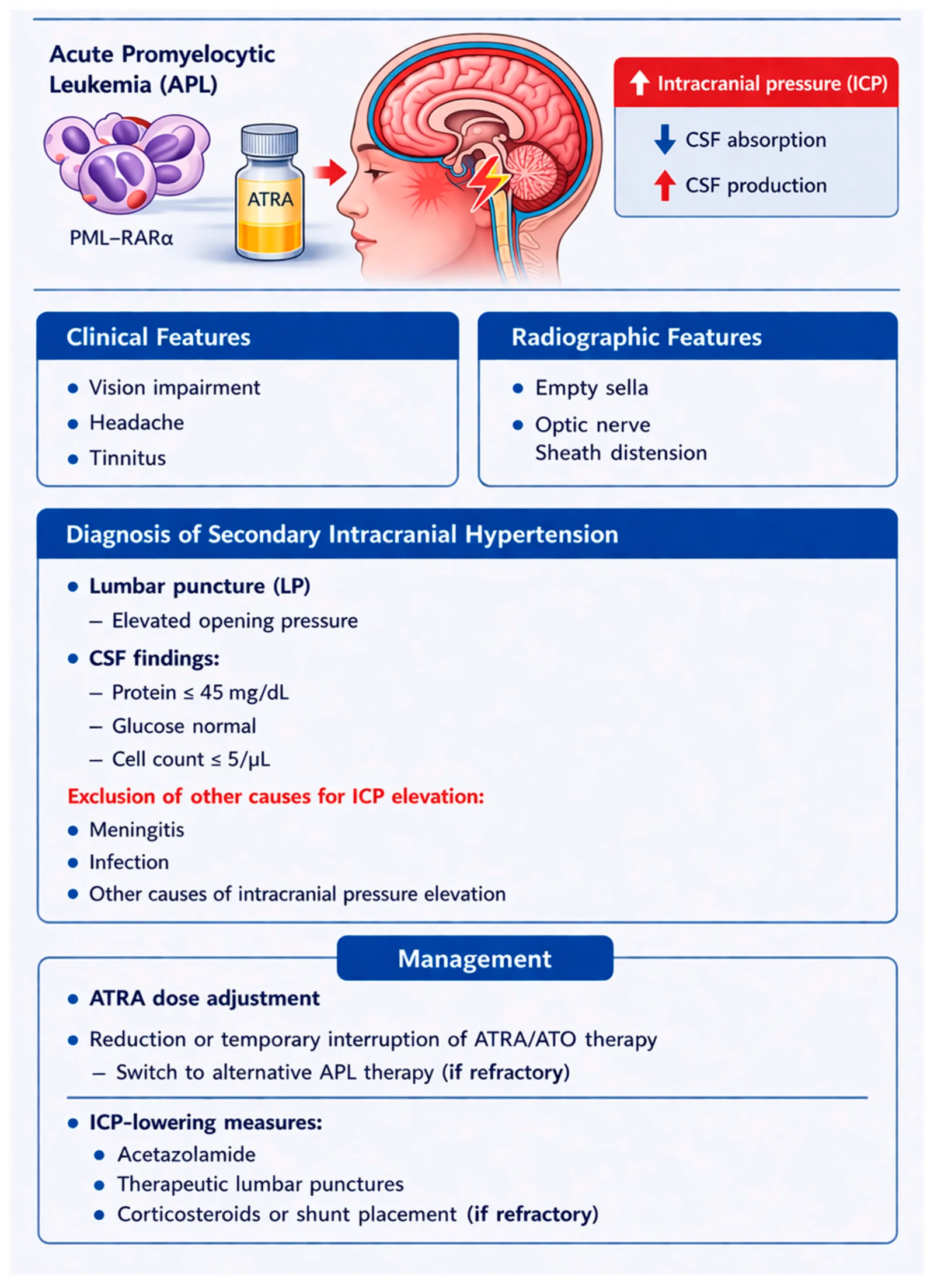
Proposed practical management framework for SIH occurring during ATRA/ATO treatment. ATRA may disrupt cerebrospinal fluid (CSF) dynamics through mechanisms analogous to hypervitaminosis A, impairing CSF absorption and possibly increasing CSF production. The resulting elevation in intracranial pressure (ICP) may present with headache, papilledema, and tinnitus despite normal structural neuroimaging. Diagnosis is supported by an elevated opening pressure on lumbar puncture with normal CSF composition and the absence of structural intracranial pathology. MRI may demonstrate indirect signs of raised ICP, such as empty sella or optic nerve sheath distension. Management includes temporary interruption and dose reduction of ATRA, ICP-lowering measures such as acetazolamide, therapeutic lumbar puncture, and, in refractory cases, treatment modification or switch to an alternative antileukemic regimen. This figure was created with the assistance of ChatGPT (OpenAI, https://chatgpt.com; accessed on 1 April 2026) using the following keywords: medical infographic; secondary intracranial hypertension during ATRA/ATO therapy for acute promyelocytic leukemia; clinical and radiographic features; diagnosis; management. The authors reviewed and edited the generated content and take full responsibility for its accuracy.

Finally, management of ATRA-associated SIH is not standardized and remains based predominantly on small series and individual case reports. The proposed management framework (Figure 2) should therefore be regarded as a pragmatic, hypothesis-generating approach rather than an evidence-based treatment algorithm.

## 4. Conclusions

ATRA-associated SIH should remain an important differential diagnosis when headache, pulsatile tinnitus, ocular pain, diplopia, focusing difficulties, or other visual symptoms develop during APL treatment, including during consolidation. Prompt neurological and ophthalmological assessment is required to identify papilledema and exclude alternative causes such as intracranial hemorrhage, cerebral venous sinus thrombosis, infection, hydrocephalus, structural lesions, and CNS leukemia.

The present case demonstrates that SIH does not necessarily require permanent discontinuation of ATRA. In selected patients, temporary interruption, therapeutic CSF drainage, acetazolamide, and cautious symptom-guided ATRA rechallenge and dose titration may permit continued differentiation therapy while controlling neurological toxicity. However, management must be individualized, and preservation of visual function remains the primary neurological priority.

The sustained molecular remission observed in our patient is reassuring but cannot establish the long-term oncological safety of reduced cumulative ATRA exposure. Larger datasets and longer follow-up are required to define optimal management, appropriate rechallenge strategies, and the long-term consequences of ATRA dose modification.

## Figures and Tables

**Table 1:** Selected reports of all-trans retinoic acid (ATRA)-associated secondary intracranial hypertension (SIH) in acute promyelocytic leukemia (APL).

Study	Patient(s)	Treatment Phase/Regimen	Management	Key Relevance
Sano et al., 1998 [9]	18-year-old man	Induction/ATRA	ATRA discontinuation, glycerol	Early adult report, onset day 23
Testi et al., 2005 [8]	Pediatric cohort (10/107 with PTC)	ATRA-based protocol therapy	Managed within pediatric APL treatment protocol	Pediatric susceptibility
Holmes et al., 2012 [12]	38-year-old woman	Induction/ATRA	ATRA discontinuation, acetazolamide, withdrawal of concomitant fluconazole	Azole antifungal-ATRA interaction as contributing factor
Smith et al., 2014 [11]	Low-/intermediate-risk APL series (5/10 developed PTC)	ATRA/ATO	ATRA interruption, acetazolamide, topiramate when acetazolamide failed	High incidence with combined ATRA/ATO, role of topiramate after acetazolamide failure
Anoop et al., 2014 [13]	25-year-old woman	Induction/ATRA	ATRA discontinuation, corticosteroids	Adult induction case, successful ATRA rechallenge
Coombs et al., 2016 [10]	ATRA-exposed cohort (4/240 probable PTC, 1.7%)	ATRA-based protocol (Intergroup 0129)	Variable: dexamethasone, or ATRA held and resumed at same or reduced dose	Cohort-level incidence, modified diagnostic criteria proposed for APL, pediatric susceptibility
Ghiaur et al., 2022 [14]	20-year-old woman, low-/intermediate-risk APL (BMI 34.5)	Consolidation/ATRA + ATO	ATRA discontinuation, acetazolamide, dexamethasone, then ATRA at 80% dose with acetazolamide	Consolidation-phase event during ATRA/ATO, successful dose-reduction strategy
Ramburuth and Khalil, 2023 [15]	30-year-old woman (BMI 40.1)	Induction/ATRA + ATO	ATRA and ATO discontinuation, two therapeutic LPs, acetazolamide, then ATRA rechallenge at 25 mg/m^2^/day with ATO resumed	Induction-phase event, transverse sinus stenosis on imaging, successful dose-reduced rechallenge
Creemers et al., 2026 [17]	19-year-old woman	Induction and consolidation/ATRA	ATRA interruption, dose reduction, acetazolamide, dexamethasone, lumboperitoneal shunt	Recurrent SIH across induction and consolidation, shunt enabled continued ATRA
Present case	31-year-old man, high-risk APL	Consolidation/ATRA + ATO	ATRA interruption/rechallenge, LP, acetazolamide, ATRA 30 mg/m^2^/day	ATRA/ATO continuation, molecular remission

Abbreviations: APL, acute promyelocytic leukemia. ATO, arsenic trioxide. ATRA, all-trans retinoic acid. LP, lumbar puncture. PTC, pseudotumor cerebri (term used by the cited study). SIH, secondary intracranial hypertension.

## Data Availability

The data presented in this case report are not publicly available due to patient privacy and ethical restrictions. Further information is available from the corresponding author upon reasonable request.
